# Dermatology

**Published:** 1895-09

**Authors:** Henry Waldo


### DERMATOLOGY.

In his Presidential address[9] before the Dermatological Society of Great Britain and Ireland, Dr. Pye-Smith said that unless pursued by scientific methods and on pathological principles, dermatology would again, he thought, become, what it once

[9] *Brit. J. Dermat.*, vol. vii., 1895, p. 219.

was, little better than a trade in cosmetics. But he holds that while "constitutional" remedies have an important place in the treatment of diseases of the skin, they are in most cases subsidiary to local applications, and that treatment in this, as in other branches of medicine, should be empirical rather than deductive. Dr. Pye-Smith considers that of all the internal physiological drugs at our disposal there are few more efficient than water. When judiciously taken in half-pint doses as a laxative in the morning, as a sedative at night, as a diuretic with a cool skin, or a diaphoretic when the skin is warm, as an expectorant or a refrigerant, its value is remarkable. He thinks no one will believe that it makes any difference whether sulphates are taken in mineral waters at Epsom, or Cheltenham, or Karlsbad, or dissolved in water by a druggist and sold in eight-ounce bottles. He thinks the contrary superstition would be as absurd as to suppose that it makes any difference in the effect of a warm bath upon the skin, whether it comes out of the earth at a given temperature, or is raised to the same degree in a kitchen boiler. But he insisted that it does make a great difference whether alkalies and salines are taken in large concentrated and infrequent doses, or in very small, very dilute, and very frequently repeated ones; and it makes a great difference whether a patient is ordered, in addition to his ordinary diet, to take three fluid ounces of water three times a day, or whether the dose be pints and quarts. In turning to what are called local remedies, Dr. Pye-Smith said we all admit the efficiency of local astringents, rubefacients, stimulants, caustics, or anodynes; but for them thus to act upon the living epithelial cells and corpuscles of the connective tissue, or the papillary blood-vessels, the touch-corpuscles, cutaneous nerves, and muscular apparatus of the hair-sacs, they must, he considered, either be absorbed through the dead horny layer of the epidermis by inunction, or they must gain access directly in aqueous solution to the living tissues when already denuded of their protecting cuticle. As he said, it is in this way that the vessels are effected by salts of lead, the sweat-glands by belladonna, and the cutaneous nerves by anodynes. He pointed out that a lotion on a whole skin runs off almost like water from a duck's back, and an ointment spread over a patch of weeping eczema never gets nearer the disease than the surface of the watery film on which it floats. Other remedies, however, those which are called ceratolytic, act, not vitally, but chemically and directly, upon the horny layer of epidermis. Others again, as sulphur, mercury, phenol, and boric acid, exert a destructive power, not on the skin itself, but on animal or vegetable invaders. Dr. Pye-Smith went on to say that there are, however, other most valuable local applications which have no such active and special properties. In fact they are chemically and physiologically inert, and yet are therapeutically useful. They act by supplying lack of sebaceous secretion, or by protecting the skin from the irritation of sweat, or urine, or dust, or cold, or heat, or even from the mere access of air at ordinary temperatures. He said we all know how important it is in applying local remedies to the skin to decide whether they shall be conveyed in the form of powder, or as an ointment, or in aqueous, alcoholic or ethereal solution ; and thought it was surely probable that the important difference of action which depends on the vehicle chosen will apply to the unmedicated vehicle itself. He said that merely covering an inflamed surface with an inert powder like starch is, we know, of great value in erysipelas and some forms of erythema and eczema; and probably similar applications of zinc, bismuth, and other chemical compounds differ rather by their physical than their physiological properties. So, also, he said, it is sometimes more important whether we choose benzoated lard, or wax and oil, or soft paraffin or lanolin for the vehicles of our ointments, than whether we put this or that drug in the vehicle. He thought we not infrequently meet with cases of dermatitis which may be properly described as *Noli me tangere*; for *any* local medication seems at once to aggravate the condition, and the only efficient treatment is to guard them sedulously from every kind of mechanical or chemical disturbance. He said he could not speak of cutaneous therapeutics without some allusion to the question of *meat* and *drink*, particularly with the modern meaning of the two words as denoting animal food and alcoholic beverages. He thinks there can be no doubt that in cutaneous medicine, as in other departments, "dieting" has been carried to an extreme and often a ridiculous minuteness. He said for most of our patients excessive quantity of food and imperfect mastication are much more important causes of illness than special quality. What most people eat is for most people wholesome, and what a natural appetite finds appetizing seldom disagrees. He believes, on most forms of cutaneous disease, diet has scarcely any effect. But he believes there are exceptions. With respect to drink, Dr. Pye-Smith thinks that water drunk freely between meals is an excellent diuretic, and seems to benefit most superficial forms of dermatitis ; and the infusion of lemon with cream of tartar, known as imperial drink, is, he thinks, an admirable refrigerant, laxative, diuretic, and sedative. He states that eczema, when very irritable, and most forms of inflammation of the skin (as of other organs) in the early stage are believed, and he thinks with good ground, to be aggravated by alcoholic beverages of any kind. But in chronic forms of eczema, in psoriasis and most other dermatoses, he is not aware of any reason for prohibiting such amount and kind of wine or malt liquor as on other considerations are found to agree with the patient ; and he is sure that in many cases of recurrent subacute erythema with anæmia, and in not a few of chronic eczema and of lupus, red wine taken with meals, and still more often porter, or, if this is not well borne, one of the lighter kinds of ales, is not only harmless but useful. He believes for the marasmus we sometimes see along with general eczema in young children there is no drug so valuable as brandy, and he believes he has seen it "cure" as unmistakably as one can desire. But Dr. Pye-Smith confesses that he does not understand why patients suffering from diseases of the skin are so frequently ordered to drink spirits and water. He says, if alcohol in some form is indicated, and there are practical reasons against the use of wine or beer, by all means let us order it. But as a routine prescription he thinks it is most unadvisable.

After Dr. Pye-Smith's address, Dr. Radcliffe Crocker read a paper on the "Internal Therapeutics of Psoriasis,"[1] in which he advocated salicylate of soda being given in fifteen-grain doses three times a day, without external medication. Dr. Crocker asked the opinion of the members present as to the efficacy of arsenic and thyroid gland in psoriasis, and the general opinion was that they were both disappointing in their effect. Arsenic was considered to be harmful if given in the early stage, or if there was much hyperæmia present, in which condition it would increase the rash. Dr. Crocker has had several cases in which the result was striking and conclusive, and the form in which this was particularly so was in extensive spreading cases of psoriasis guttata of recent development, the very form which is usually unsuited both for thyroid extract and for arsenic, the former especially sometimes producing a very rapid extension and multiplication of the patches in this class of case. In cases where there were only a few chronic patches the result was not, as might be expected, so brilliant. Dr. Crocker explained that the first effect of the drug appears to be the diminution of hyperæmia, so that the patches become paler, the scales are no longer formed abundantly, and the old scaly crusts are detached or are easily detachable, exposing a pale red surface, which gets smoother week by week, and finally leaves only a slightly stained surface. Dr. Crocker went on to relate what drawbacks he had found. In the more chronic forms of the disease he said the improvement had not been so striking, though, except in a few very old patches, there had nearly always been marked improvement. A few of his patients were unable to continue salicylate of soda, as it produced dyspepsia in the form of pain in the epigastrium soon after taking it. It was always given after meals to obviate any gastric irritation as far as possible. I find between meals is a better time to give salicylates, and it then appears to cause little or no nausea. It has been recently suggested that giving it in cinnamon water obviates any tendency to nausea. Salicin agrees better with some patients. It is possible that the pure natural salicin or salicylate may

[1] *Brit. J. Dermat.*, vol. vii., 1895, p. 227.

be more easily tolerated, but in the great majority of cases the cheaper synthetical product is sufficiently efficacious. Dr. Crocker has found salicylate beneficial in some other diseases of the skin,—for instance, in various forms of erythema multiforme, including erythema iris,—and he especially suggested its administration in erythema nodosum.

Mr. Jonathan Hutchinson reports[1] a case illustrating the antecedents of lupus vulgaris in a middle-aged man. Mr. Hutchinson is no believer in the inoculation of the tubercle bacillus from without, as an ordinary cause of lupus. It seems to him far more probable that the parasite exists during long periods in a state of latency, from which any local injury may rouse it into activity. In this instance he thinks it was the occurrence of a boil which, reducing the vitality of the skin, made it an easy prey to the parasite.

Mr. Jonathan Hutchinson[2] says that he ventures with some confidence to propose that the adjective "herpetiformis" should be used in reference to those forms of morphœa which resemble herpes in their distribution. He feels sure that this word will be found convenient, and that it will denote an obvious and important clinical fact. He mentions the following case, which he considers affords an excellent illustration of what he means: The group of morphœa spots were arranged in two long oval leashes on the clavicular region, exactly like herpes. The patches were also made up of separate spots, which, although they had coalesced in some instances, had by no means done so in all. Mr. Hutchinson considers the term "herpetiform" may be suitably applied to all cases of morphœa in which the changes are arranged in bands, or after the manner of a corymb or panicle. More than a few of these cases will be found to be bilateral, and in a certain number the streaks may be arranged almost symmetrically. Mr. Hutchinson goes on to say that the tendency to be bilateral, and to occur on many parts at once, is very rare in zoster, and very common in morphœa, but, in his opinion, does not in the least invalidate the theory of the nerve distribution of the latter. It proves only that many nerves may be affected at the same time, and possibly that in some cases there is a definite tendency for the same nerve-trunk on the two sides to suffer. Mr. Hutchinson hopes he will not be blamed for preferring still to use the old name "morphœa," rather than the new one of "sclerodermia." The latter, he says, is a pathological term; it denotes a theory, and is applicable to many affections, and to only certain stages of each. The former is a clinical one, is well understood in its conventional signification, and has

[1] *Clin. J.*, vol. vi., 1895, p. 172. [2] *Brit. M. J.*, 1895, vol. i., pp. 1194, 1434.

the great advantage of being almost meaningless as regards pathological theory. He says that, using the word morphœa in its old sense as applicable to all hide-bound conditions of skin, he would propose, by suitable adjectives, to discriminate its different forms. For clinical purposes these latter present us with most important pecularities of character. Mr. Hutchinson suggests that we may perhaps conveniently recognise three principal divisions, under each of which several groups may in the future be arranged. They are the following:

*Division* I.—Herpetiform morphœa; the patches being arranged on the pattern of herpes in streaks or bands, and however extensively bilateral, seldom or never with exact symmetry.

*Division* II.—Acroteric morphœa, in which, although the whole skin may suffer more or less, the beginning and the greatest severity of the disease are almost invariably at the extremities. No location in streaks, symmetry always marked, and Raynaud's phenomena not unusual. This form is met with almost solely, Hutchinson thinks, in adult women, and shows no tendency to spontaneous cure.

*Division* III.—Hide-bound conditions of skin, in which the subcutaneous tissue rather than the skin itself is implicated. In this division are to be placed for the present certain cases which differ more or less definitely from both the preceding, and concerning the exact relations of which it is very difficult to speak. There are no ivory patches, nor are the extremities preferentially affected.

Dr. Allan Jamieson[1] says there are many who have the idea that cutaneous disorders are necessarily due to dirt. He asks us to discard this opinion. For if the laity hold that scabies is essentially due to dirt, they are right if they are considering the relation between the sum total of dirt and the sum total of this disease; but they are wrong if they wish to show that there is direct connection between the scabies of an individual and his personal habits of cleanliness. The most cleanly person may have the disease communicated to him; then do what he may in the ordinary domestic routine of bathing and washing, he remains infected until medical aids are brought to his assistance. He will never spontaneously get well, a fact we may remember by associating with it the saying of Dr. Leloir, that some people are born (or infected shortly after birth) with scabies, live their lives with scabies, and die in the same company. Dr. Jamieson warns us against the belief which seems to be commonly held amongst the members of the medical profession, that arsenic is a specific in skin diseases. Arsenic, he considers, is not such a specific, and is not by any means to be prescribed indiscriminately. The drug

[1] *Clin. J.*, vol. vi., 1895, p. 72.

possesses undoubted effects on the skin, as is evidenced by the disappearance of certain eruptions under its use, those of lichen planus, pemphigus, dermatitis herpetiformis, and, in some cases, psoriasis. That arsenic may cure or cause pathological conditions of the skin is shown by the fact that its long-continued administration produces hyperidrosis of the feet, a peculiar keratosis of warty nature of the soles and palms, and occasionally a species of cancer and well-marked pigmentation. Dr. Jamieson says we shall do well to remember once for all that it is absolutely useless in eczema. Dermatologists, however, differ in this view, as I recently heard Dr. Robert Liveing say at a meeting of the Dermatological Society that it was very valuable in some forms of eczema. Dr. Jamieson advises that the treatment of skin diseases should be carried out exactly on the same principles as is that of the diseases of other parts. Rest, he considers, is of primary importance; rest, together with the removal of all causes of irritation. Then soothing treatment, and only cautious—very cautious—stimulation.

In the Dermatological Section of the recent Meeting of the British Medical Association, Professor McCall Anderson opened a discussion on the pathology and treatment of pruritus. Dr. Anderson thinks the itching must be treated empirically. He prefers the employment of electricity, atropia subcutaneously, or the coal-tar derivatives, such as antipyrine and phenacetin, in gradually increasing doses.

Dr. H. G. Brooke followed with a paper on the same subject. He thought the internal cause of pruritus should include the purely *nervous* pruritus—the primary pruritus of French authors. The tendency of the French school to deal much in "nerves" was alluded to. He considered it to be a "diathesis of itching," demanding external manifestation, and accompanied not infrequently by internal metastases, such as hay fever. Other forms of nervous itching, the expression of organic nerve disease, alienated mental states due to reflex causes, were also placed in this division as well as the influence of various poisons in the blood—*e.g.*, in gout, jaundice, diabetes, rachitis, foods, and drugs. The external cause included local skin diseases and parasitic skin diseases, as well as the pruritus due to changes in temperature and alterations in blood pressure. These were frequently the causes of irritation of the skin. He thought that its treatment could be properly undertaken only when the underlying causes were taken into account.

Of the numerous patients exhibited in London, I thought the most interesting were those shown by Mr. George Stoker. The patients suffering from alopecia areata wear an indiarubber "chimney pot" hat, which is filled with pure oxygen through a tube communicating with it. One of them was a girl aged 8, whose head became bald in August, 1894. Before this treatment there was a slight downy growth on the left occiput, and a few straggling hairs on the right parietal regions and on left eyebrow. After treatment upon this plan for two months there was growth of hair one-and-a-half inches long all over the head.

That oxygen possesses the power of healing very extensive ulceration, is shown by the case of a girl aged 16, who, when oxygen was ordered, had an ulcer on back (following burn), eleven inches by nine inches. Ulcer now measures five inches by three-and-a-half inches.

Special attention should be given to a paper by Dr. Galloway on "The Nature and Causation of the Skin Lesions occurring in Nervous Diseases."[1]

HENRY WALDO.

### Reviews of Books.

[1] *Brit. J. Dermat.*, vol. vii., 1895, p. 304.

